# Two-dimensional CNN-based distinction of human emotions from EEG channels selected by multi-objective evolutionary algorithm

**DOI:** 10.1038/s41598-022-07517-5

**Published:** 2022-03-03

**Authors:** Luis Alfredo Moctezuma, Takashi Abe, Marta Molinas

**Affiliations:** 1 Department of Engineering Cybernetics, Norwegian University of Science and Technology, 7491 Trondheim, Norway; 2 International Institute for Integrative Sleep Medicine (WPI-IIIS), University of Tsukuba, Tsukuba, Japan

**Keywords:** Learning algorithms, Emotion, Computational science, Computational neuroscience, Data processing, Machine learning

## Abstract

In this study we explore how different levels of emotional intensity (Arousal) and pleasantness (Valence) are reflected in electroencephalographic (EEG) signals. We performed the experiments on EEG data of 32 subjects from the DEAP public dataset, where the subjects were stimulated using 60-s videos to elicitate different levels of Arousal/Valence and then self-reported the rating from 1 to 9 using the self-assessment Manikin (SAM). The EEG data was pre-processed and used as input to a convolutional neural network (CNN). First, the 32 EEG channels were used to compute the maximum accuracy level obtainable for each subject as well as for creating a single model using data from all the subjects. The experiment was repeated using one channel at a time, to see if specific channels contain more information to discriminate between low vs high arousal/valence. The results indicate than using one channel the accuracy is lower compared to using all the 32 channels. An optimization process for EEG channel selection is then designed with the Non-dominated Sorting Genetic Algorithm II (NSGA-II) with the objective to obtain optimal channel combinations with high accuracy recognition. The genetic algorithm evaluates all possible combinations using a chromosome representation for all the 32 channels, and the EEG data from each chromosome in the different populations are tested iteratively solving two unconstrained objectives; to maximize classification accuracy and to reduce the number of required EEG channels for the classification process. Best combinations obtained from a Pareto-front suggests that as few as 8–10 channels can fulfill this condition and provide the basis for a lighter design of EEG systems for emotion recognition. In the best case, the results show accuracies of up to 1.00 for *low vs high arousal* using eight EEG channels, and 1.00 for *low vs high valence* using only two EEG channels. These results are encouraging for research and healthcare applications that will require automatic emotion recognition with wearable EEG.

## Introduction

Human communication, our social structure, personal life, mental and even physical health are mainly governed by emotions, which are generated, regulated and guided by our brain^1–3^. Emotion is a psycho-physiological expression/phenomena that reflects survival-related functions/circuits supposedly connected in our brain by evolution, which can be present across species/cultures^4–8^. An emotion is triggered as response to imaginary/real stimuli, conscious/unconscious perception of an object or situation, and it is often associated with *subject’s thinking, behavioral responses*, and *a degree of pleasure or displeasure*^4,5,7,9–13^.

Emotions can be expressed *verbally* and *non-verbally*; *verbally*, associating a word with an emotion using different intonation of voice^14,15^, and *non-verbally* with physiological reactions (i.e heart pounding, cold hands, sweating or turning red, which are the result of a subcortical amygdala-based system), facial expressions, body language, gestures and feelings^2,5,15,16^. Emotions with meaning and cognitive content are the result of reciprocal connections between the subcortical emotions system and the medial prefrontal cortex, and feedback and control of the prefrontal cortex in the amygdala^17,18^.

There are two well-accepted emotion classification models: (1) Basic emotion-based classification, which argues that there are several basic emotion types, for instance some works propose that 6 or 11 basic emotions types are combined to form all human emotions^19–22^. (2) Dimensional space-based classification, which holds that emotions are not discrete, and that the similarity and difference between emotions are represented according to their distance in the dimensional space. This model proposes that all affective states arise from two fundamental neurophysiological systems, one related to valence (a pleasure-displeasure continuum) and the other to arousal, or alertness (related to the perceived intensity of an event). In the dimensional space-based model, these 2-D spatial model combined by *Arousal* and *Valence* is the most used^2,22–26^.

Verbal emotional expressions, and some of the non-verbal emotions can be distinguished or recognized by analyzing facial expressions, voice tone, feeling the cold hands, etcetera. However, these signs are easy to be disguised or camouflaged^27^. There exist computational approaches to automatically recognize/classify emotions from different data sources such as cerebral blood flow, electrooculography (EOG), electrocardiogram (ECG), blood volume pulse, galvanic skin response, respiration, phalanx temperature, and brain signal collected by electroencephalogram (EEG)^2,15,28–32^.

Emotion recognition can be useful for a variety of different domains and EEG-based applications including education, security, mental health and general healthcare. It can be used by school teachers to monitor behavior related to Autism Spectrum Disorder (ASD) and quickly identify important events; this information can then be analyzed by experts and health professionals^18,33^. With an emotion model based on *Low vs High Arousal/valence* classification one can create a two-dimensional plot in real-time showing the emotion response to stimulus, which can be questions during a job interview where it is required to determine reliability traits^31^. It can be applied to medical care to recognize the emotional state of patients with expression disorders, and this can help to make different nursing measures according to the patients’ emotions and improve the quality of nursing^26,34^.

Different approaches for EEG-based emotion recognition have been proposed, and current public datasets include at least self-reported emotions using *Arousal* and *Valence* from emotional videos/film clips, and the most used datasets are: DEAP, IAPS, Mahnob HCI-Tagging, SEED, SEED-IV, and DREAMER^2,35–41^.

Although different public datasets have been studied and proposed methods for EEG-based emotion recognition, the results obtained with different datasets are not comparable, since most of the public datasets are not using a standardized set of stimulus. To measure self-reported emotions, the Self-Assessment Manikin (SAM) is widely used. It is a non-verbal, picture-oriented questionnaire technique that can measure the pleasure, *Arousal*, *Valence*, and dominance associated with a subject’s affective reaction to a wide variety of stimuli^42^.

When it comes to feature extraction and classification, Deep Learning (DL)-based approaches have shown to be a success in image processing, and other fields, but when working over EEG datasets they have not shown better results in most of the proposed architectures compared with classical machine learning (ML) approaches such as Support Vector Machine (SVM), naive Bayes, random forest, etcetera^43–46^. DL architectures generally requires a large amount of data, which is not common when working with EEG data, as collecting a large number of instances or for long periods of time is unrealistic.

Recently, Convolutional Neural Network (CNN) architectures have been proposed for different EEG-based classification tasks, for example, a CNN Gated Recurrent Unit (CNN-GRU) was tested for subject identification in the DEAP dataset using the 32 subjects and 10-second segments of EEG signals. The authors reported that using CNN-GRU it is possible to reach up to 0.999 mean Correct Recognition Rate (CRR) for subject identification using 32 channels, and 0.991 with 5 channels that were selected using one-way repeated measures ANOVA with Bonferroni pairwise comparison^47^. The task tested with the CNN-GRU is not for emotion recognition, but it was shown that CNN can be used in the DEAP dataset. Another recent proposed CNN architecture is the EEGNet, which has been tested for different EEG-based task classification, and it has shown higher accuracies than some ML-based classifiers^43,48,49^.

Another approach which combines CNN, Sparse Autoencoder (SAE), and Deep Neural Network (DNN) was tested in the DEAP and SEED datasets^50^. In the case of DEAP, the highest recognition accuracies for valence and arousal were 89.49% and 92.86% respectively, and 96.77% for the SEED dataset. It should be noted that the results are not comparable since DEAP and SEED datasets contain different number of subjects, classes, instances, and evaluation (DEAP: arousal and valence, and SEED: negative, positive, neutral).

Published works report the use of simple Neural Networks (NN) structures such as using a single hidden layer structure, and the use of recurrent and convolutional neural network (RNN and CNN). However, to train the most complex cases it is required to improve the computational power with faster Central Processing Units (CPUs) and/or the use of Graphics Processing Units (GPUs)^43,47–49^.

DEAP dataset has been used in different proposed methods, for instance, there is a work where authors used the Discrete Wavelet Transform (DWT) to decompose the EEG signals and use the coefficients of decomposition 3 for computing a set of statistical values. Lastly, Principal Component Analysis (PCA) is applied for reducing the set of features^51^ and thus use SVM for classification. The results reported shows that using that approach the most relevant channels are *PO4* and *AF4* obtaining accuracies above 80%.

In this paper, we first compare our previous proposed method based on DWT for feature extraction, which were robust for extracting features for epileptic seizure classification and EEG-based biometric systems^52–57^. In the experiments exposed here, we first use the 32 EEG channels of the DEAP dataset, and three different classifiers also based in our previous research; SVM, naive Bayes (NB), and k-Nearest Neighbors (kNN).

We then explored the use of CNN through the EEGNet architecture, testing first the use of all the EEG channels available for *Low vs High Arousal* and *Low vs High Valence* classification. For this, we compared the performance creating one model per subject and one single model (i.e using the data from all the subjects for the same model). This experiment was repeated testing the use of segments with different duration (2, 5, 10 s) from which we found that 2-s segments works better. We then performed a set of experiments to analyze the accuracy that can be reached if we use EEG data from only one EEG channel, and this was repeated for all the 32 EEG channels.

Additionally to the feature extraction, an important step for decreasing the computational cost of any DL/ML algorithm is the selection of the most relevant channels. The EEG channel selection process is in itself informative because it can provide information about the most relevant areas in the brain for a certain neural task for a certain subject or group of subjects. This can be analyzed using apriori information related to the paradigm, which can limit the search space and therefore the results^57^.

With a well-defined automatic method for channel selection we can extract the most essential information from a minimum set of EEG channels and thus reach cheaper low-density EEG headset, as well as task-specific channel combinations. Selecting a set of channels will allow us to focus on the most relevant information or brain area, and with this decrease the computational cost for real-time processing and selecting the correct channels contribute to increase the classification performance. Additionally, these techniques will enable cheap home EEG devices that can facilitate long-term monitoring in daily life not limited to hospital/laboratories service^57^.

For tacking the channel selection problem we applied the non-dominated sorting genetic algorithm II (NSGA-II) for optimizing two objectives: (1) maximize the accuracy obtained for *Low vs High Arousal* or *Low vs High Valence* classification, and (2) minimize the number of EEG channels used for achieving (1). We selected NSGA-II because it has shown to be robust in dealing with two-objective optimization problems^53,57–59^.

Given the characteristics of the experiments exposed and the use of CNN, we performed all the experiments for this study using GPUs on the NTNU IDUN computing cluster^60^. The cluster has more than 70 nodes and 90 general-purpose GPUs (GPGPUs). Each node contains two Intel Xeon cores and at least 128 GB of main memory and is connected to an Infiniband network. Half of the nodes are equipped with two or more Nvidia Tesla P100 or V100 GPGPUs. Idun’s storage is provided by two storage arrays and a Lustre parallel distributed file system.

## Results

### DWT-based feature extraction and ML for low vs high arousal/valence classification

We tested a previously proposed method for feature extraction based on DWT with four decomposition levels, and for each sub-band extracted, the Teager and instantaneous energy, Higuchi and Petrosian fractal dimension features were computed, obtaining thus $5*4=20$ features for each EEG channel^52–56^. The obtained features from all the EEG channels were used as input for *SVM*, *NB* and *kNN* classifiers using 10-fold cross validation to compute the accuracy.

In order to identify if the process works better using a specific EEG signal segment size, we experimentally defined four EEG signal segments to be tested using the feature extraction and classification process briefly described above.

Firstly, we have tested the use of the 60 s of the video (total duration of each video), however the number of instances per subject is low, and in some cases it is not enough for the 10-fold cross validation. We have also tested the process extracting segments of 10, 5 and 2 s per video. The total number of instances per subject for Arousal and Valence, is presented in Table 1, which corresponds to both low and high Arousal/Valence. For example, for subject 1 and using 60-s segments the number of instances is 38, which corresponds to 19 low and 19 high Arousal instances. The number of instances per subject was carefully analyzed to obtain balanced datasets, selecting the lower number of instances for low or high Arousal/Valence, i.e if we have 19 and 25 instances for low and high Arousal respectively, we have selected 19 instances for each class.

**Table 1:** Number of instances per subject from the DEAP dataset, using different EEG signal segment sizes.

Subject	60 s		10 s		5 s		2 s	
	Arousal	Valence	Arousal	Valence	Arousal	Valence	Arousal	Valence
1	38	32	228	192	456	384	1140	960
2	30	32	180	192	360	384	900	960
3	36	16	216	96	432	192	1080	480
4	32	32	192	192	384	384	960	960
5	32	38	192	228	384	456	960	1140
6	20	34	120	204	240	408	600	1020
7	24	30	144	180	288	360	720	900
8	36	32	216	192	432	384	1080	960
9	38	30	228	180	456	360	1140	900
10	40	36	240	216	480	432	1200	1080
11	32	30	192	180	384	360	960	900
12	38	14	228	84	456	168	1140	420
13	36	12	216	72	432	144	1080	360
14	40	26	240	156	480	312	1200	780
15	40	38	240	228	480	456	1200	1140
16	30	40	180	240	360	480	900	1200
17	36	30	216	180	432	360	1080	900
18	28	30	168	180	336	360	840	900
19	34	26	204	156	408	312	1020	780
20	34	18	204	108	408	216	1020	540
21	38	16	228	96	456	192	1140	480
22	40	30	240	180	480	360	1200	900
23	26	26	156	156	312	312	780	780
24	38	14	228	84	456	168	1140	420
25	38	20	228	120	456	240	1140	600
26	28	34	168	204	336	408	840	1020
27	20	26	120	156	240	312	600	780
28	30	40	180	240	360	480	900	1200
29	34	26	204	156	408	312	1020	780
30	26	40	156	240	312	480	780	1200
31	28	40	168	240	336	480	840	1200
32	40	24	240	144	480	288	1200	720
All			6672	6312	13344	12624	33360	31560

As it is shown in Table 1, for some subjects the number of instances is lower than 10 when using 60-second segments, therefore the k-fold cross validation was changed accordingly in each case. With the obtained DWT-based features, we created an ML-based model per each subject. We have tested three classifiers which obtained the highest accuracies for different tasks in our previous research^52–56^.

The first classifier used was the well-known SVM, as it provides a global solution and the classification complexity does not depend on the feature dimension^61^. For SVM, the kernels tested are sigmoid, linear, and radial basis functions (RBFs). The second classifier was the k-nearest neighbors (KNN) classifier, with *1–9* neighbors. Finally, the naive Bayes (NB) classifier was also tested to analyze its performance for this task. The implementation of each classifier internally selects the best parameters by testing the set of possible parameters in each case, for instance, KNN was tested with 1–9 neighbors, but the number of neighbors used in the final classifier was the one with the highest accuracy.

The experiment consists of creating one ML-model per subject using 10-fold cross-validation and presents the average accuracy and standard deviation across the 32 subjects. Table 2 presents results obtained using the different EEG signal segments, for both, *low and high Arousal* classification, as well as *low and high Valence* classification.

**Table 2:** Classification accuracy for low vs high arousal/valence using DWT-based feature extraction using 32 EEG channels, proven with three classifiers and different signal segment sizes.

Segment (s)	Low vs. high arousal			Low vs. high valence		
	SVM	NB	kNN	SVM	NB	kNN
60	0.658 ± 0.23	0.629 ± 0.25	0.687 ± 0.23	0.571 ± 0.27	0.577 ± 0.28	0.645 ± 0.25
10	0.638 ± 0.15	0.605 ± 0.16	0.629 ± 0.13	0.614 ± 0.15	0.574 ± 0.17	0.605 ± 0.15
5	0.621 ± 0.13	0.599 ± 0.14	0.606 ± 0.19	0.608 ± 0.13	0.568 ± 0.15	0.600 ± 0.12
2	0.605 ± 0.11	0.586 ± 0.12	0.590 ± 0.09	0.584 ± 0.11	0.561 ± 0.13	0.574 ± 0.10

The DWT-based method for feature extraction has been previously used for different EEG-related task classification, however, as it is presented in Table 2, for *low and high arousal/valence* classification, all the accuracies obtained are around the level of chance for two classes, which is 50% or 0.500.

Up to now, most of the DL-based approached proposed in the literature have been not shown convincing or better results than using ML-based models^43–46^. However, the EEGNet has been tested for different EEG-based task classifications, exhibiting higher accuracies than some ML-based classifiers^43,48,49^. Taking advantage of the smaller EEG signal segments we increase the number of instances for training and testing the models, and by doing so circumvent the issue of large amount of data required by EEGNet^62^. We experimentally found that EEGNet-based models can be successfully used for low and high arousal/valence classification. This is explained and presented in the following experiments.

### Exploring the number of Epoch for training the EEGNet models

As it is presented in Table 1, when using 60-second segments, the number of instances per class is low, and it cannot be used for training a neural network, separating the dataset for each subject on (1) training ($50\%$ of the data), (2) validation ($25\%$) and (3) test ($25\%$) sets. Therefore, in the following experiments, we have only considered EEG signal segments of 10, 5 and 2 s.

We run 300 epochs or training iterations using the EEG raw data after pre-processing from all the channels, and each subject separately, saving the model weights that produced the highest accuracies. Experimentally we found that, for all subjects, when increasing the number of epochs to around 150-200, the training and validation accuracies becomes nearly 1.000, and after that there are some fluctuations but it remains similar.

**Figure 1:**
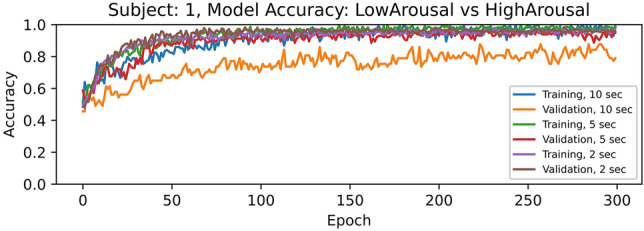
Accuracies for *low arousal* vs *high arousal* classification using segments of 10, 5, and 2 s from all the EEG channels of subject 1.

**Figure 2:**
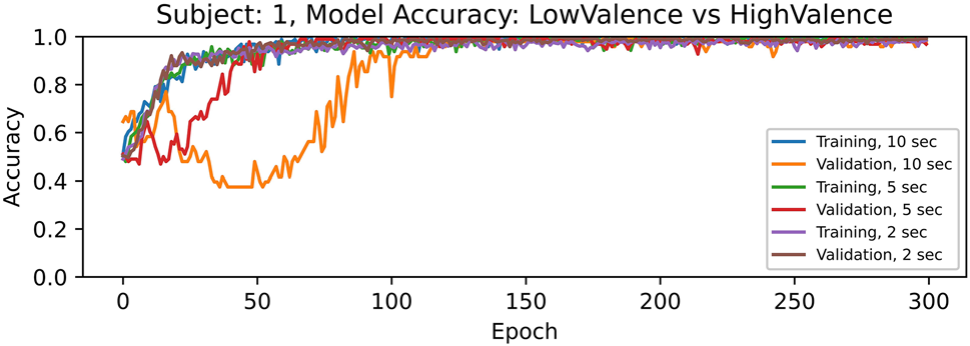
Accuracies for *low valence* vs *high valence* classification using segments of 10,5, and 2 s from all the EEG channels of subject 1.

To illustrate the aforementioned behavior, Figs. 1 and 2 present the results using EEG signal segments of 10, 5 and 2 s using all the channels from Subject 1, for low vs. high arousal and valence, respectively.

We noticed that when EEG signal segments of 10 or 5 s are used, the training accuracy increases slightly more and faster than with 2 s. However, the validation accuracies are lower. Based on these findings, we considered 200 epochs for training the models in the subsequent experiments.

Once we identified the candidate number of epochs to be used for training the EEGNet, we repeated the experiments to analyze its performance for classifying *LowArousal vs HighArousal* and *LowValence vs HighValence* using all the EEG channels and different EEG signal segments. This is relevant, since when we extract smaller EEG signal segments, we use more instances for training, validating and testing the created models (see Table 1).

**Figure 3:**
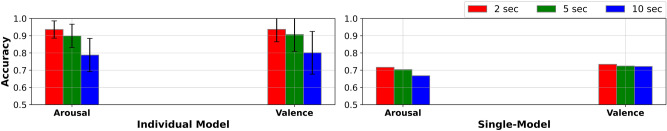
Left: Average accuracies and standard deviation obtained with EEGNet per each subject for *low vs high arousal/valence* classification using all the EEG channels from 2, 5 and 10-s signal segments. Right: Accuracies obtained with a single EEGNet model with data from all the subject for *low vs high arousal/valence* classification using all the EEG channels from 2, 5 and 10-s signal segments.

We run the classification process with EEGNet for each subject, and for each of the three EEG signal segments, one subject at a time. The average accuracies obtained and the standard deviation across subjects is presented in left part of Fig. 3. The results show that using 2-s segments, the higher accuracies are reached, and also the standard deviation is lower across subjects.

Following the same process, the question about a possible single model from all subjects naturally comes up, since the accuracies obtained creating an individual model per subject are higher when using 2-s segments (i.e higher number of instances). To investigate this, we have created a single model for all subjects to classify low vs high Arousal, and low vs high Valence using also 2, 5 and 10 s, but now using the instances from all the subjects. This model using the three different EEG signal segments shows the average accuracies presented in right part of Fig. 3.

It should be noted that the number of instances presented in Table 1 is not the sum of the instances from all the subjects, since that number is modified in the process of balancing the dataset. For instance, using 10-s segments and Arousal, if we sum the instances used for each subjects the total is 6360, however the number of instances used in the DL-model using instances from all the subjects is 6672. This is because as it was explained previously, the dataset was carefully balanced for each subject, and for the single model, all the instances from all the subjects were organized first and the dataset was balanced at the end, which allows the use of 312 more instances (i.e 156 for each low and 156 for high arousal).

Figure 3 has shown that creating one model per subject using 2-s segments, the higher accuracies can be reached. It also shows that when creating a unique model with data from all the subjects, the highest accuracies are also reached using 2-s segments, however the accuracy is around $25\%$ lower than creating a model for each subject.

Based on the results obtained, it is clear that the highest accuracies are obtained creating a model for each subject, therefore for the rest of experiments, we will consider only this approach. Additionally, we analyze whether there exist a small set of optimal EEG channels for obtaining the same or higher accuracies than these.

### Using a single channel for *low vs high arousal/valence* classification

The objective of the current set of experiments is to investigate the accuracies obtained using EEGNet, creating one model per subject, but instead of using all the channels, here we will use only one EEG channel at a time. For comparison purposes, we have repeated the experiments using one channel at a time, for all the subjects, and the three EEG signal segments (i.e 2, 5 and 10-s segments). For example, we created a DL-model for each subject using EEGNet and EEG data from channel *Fp1* only. Then, we calculated the average accuracy and the standard deviation, which in the case of *low vs high arousal* are 0.633±0.07, 0.636±0.08, 0.601±0.10, using 2, 5, and 10-s segments respectively. In this way, we can analyze if there exist a specific EEG channel that works better for all the subjects, and also to compare the accuracy using different EEG signal segments.

This analysis is relevant since in recent published works^12^, the authors argue that in the best case, using only EEG channel *C*3 for *low vs high arousal*, they can obtain an accuracy up to 91.07%. They also argue that using *Oz* they can achieve up to 98.93% of accuracy for *low vs high valence*.

**Figure 4:**
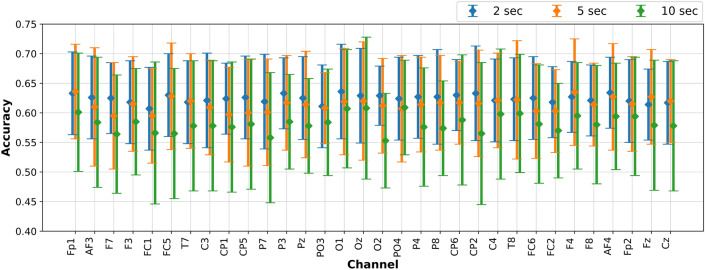
Low vs high arousal classification accuracies and standard deviation for the test set (25% of the data) using EEG data from one channel at a time and the EEGNet.

**Figure 5:**
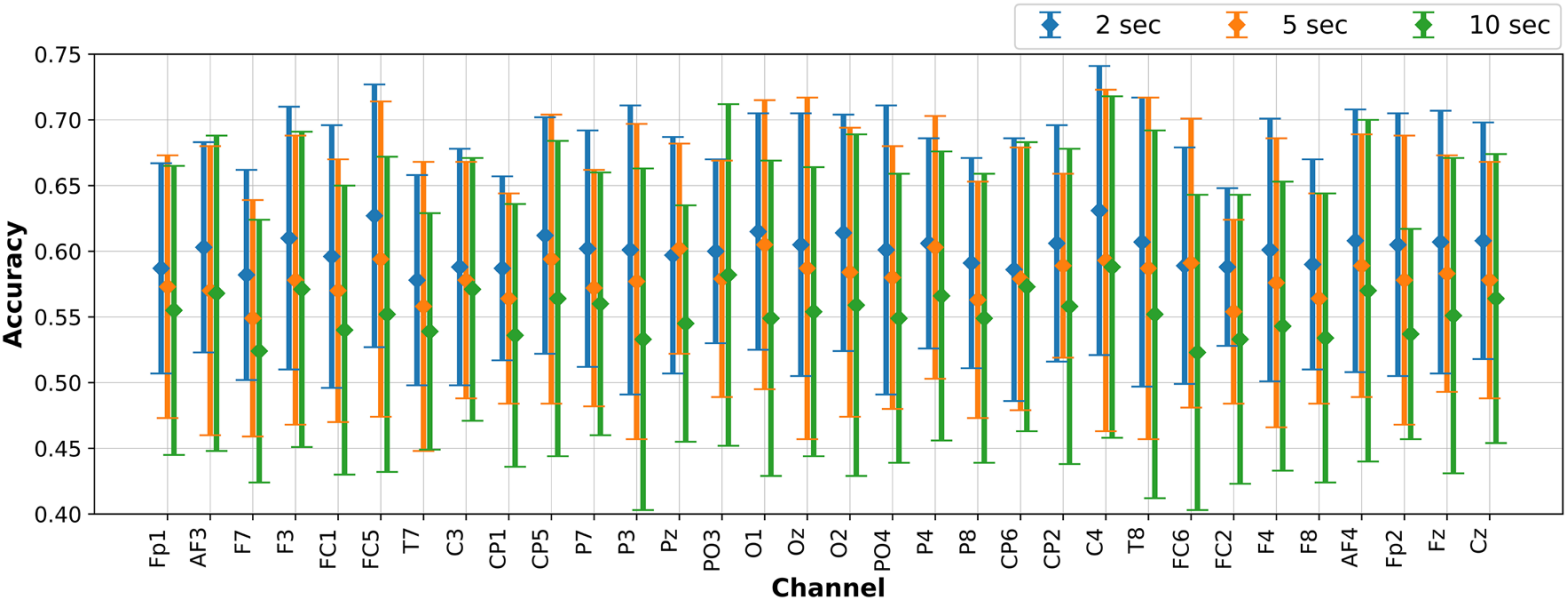
Low vs high valence classification accuracies and standard deviation for the test set (25% of the data) using EEG data from one channel at a time and the EEGNet.

The average accuracies and standard deviation obtained from all the subjects and using EEG data from one channel at a time, are presented in Figs. 4 and 5 for *low vs high arousal/valence*, respectively.

The aforementioned work^12^ used a different process and different pre-trained CNN, which does not allow a consistent comparison of results. Looking at the best channels selected by them (*C3 and Oz*), and our results, we did not find similar results as claimed by them, since our accuracy results are lower and also the channels for obtaining the highest accuracies are different (see Figs. 4 and 5). However, examining the higher accuracy single channels, hints on that certain channel combinations per subject (individually), may increase the accuracy if an optimization approach is implemented. This has been shown to work so in other tasks^53,57,59^.

### Optimized EEG channel reduction and selection for *low vs high arousal* classification

As it has been shown in the previous experiments, the accuracies are higher when using 2-second segments, this may be related to the number of instances available for training the EEGNet models. The general configuration of the experiment consisted on *low vs high arousal* classification using 2-s segments of EEG signals, creating one model per subject. In this section, the process was repeated several times trying to identify if the accuracy increases using different channel sub-sets. For this we have designed and implemented an optimization process with the NSGA-II.

In short, NSGA-II uses a binary chromosome representation of 32 genes, one gene per EEG channel, and each gene with two possible values; 1 if the channel is used, 0 if not. The optimization algorithm generates chromosome populations that are evaluated based on the highest accuracies and the ones with the highest are re-used to generate new populations. To select the best chromosomes in each population, the algorithm uses two metrics that are optimized: the number of channels must be concurrently as low as possible, and the accuracy as high as possible.

**Figure 6:**
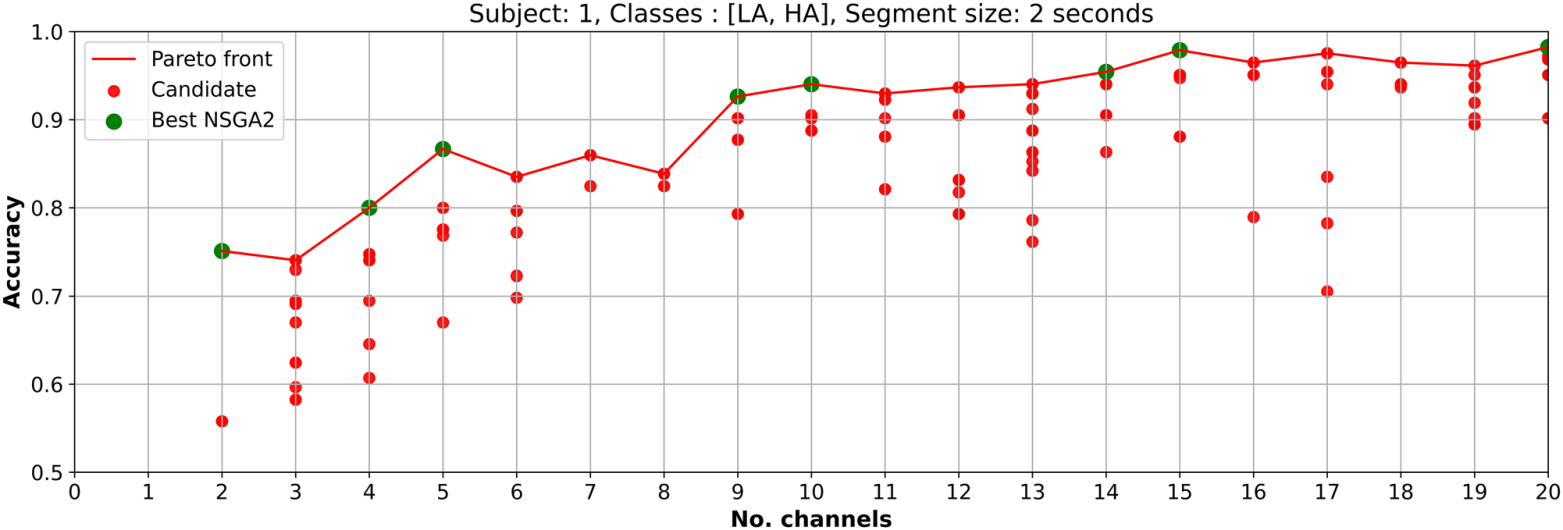
Optimized EEG channel selection results for low and high arousal classification using EEGNet and data from subject 1.

**Figure 7:**
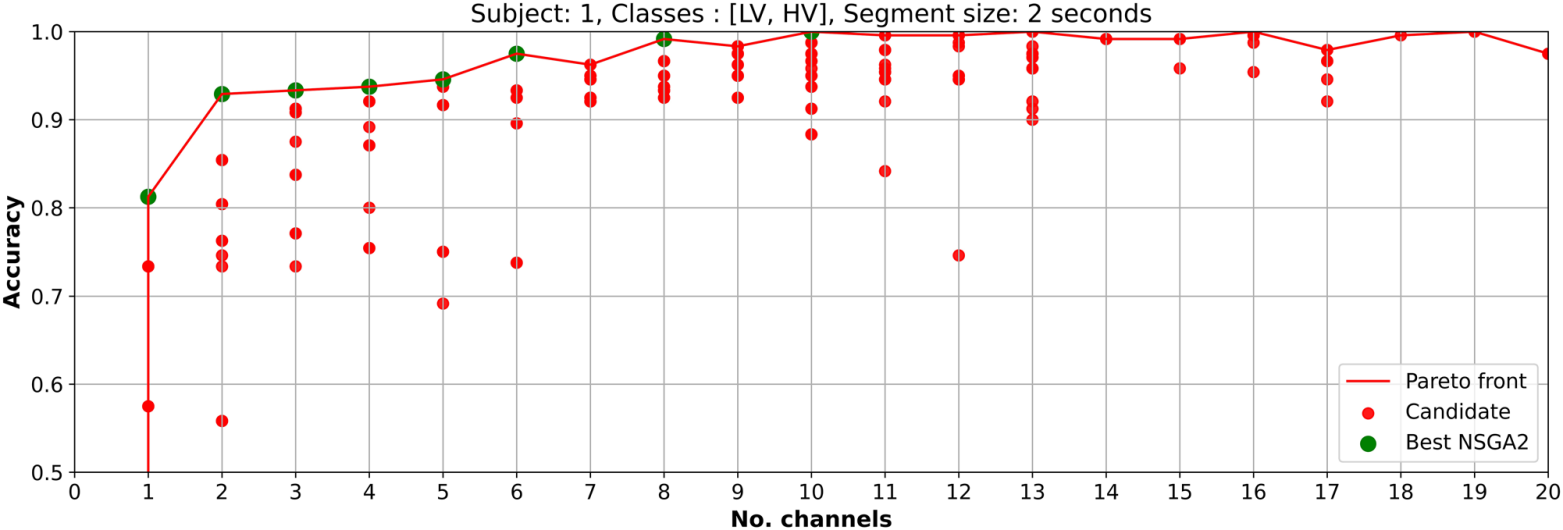
Optimized EEG channel selection results for low and high valence classification using EEGNet and data from subject 1.

Figures 6 and 7 present the the optimization process of subject 1 for *low vs high arousal/valence* classification, respectively, using EEGNet and the channel selection process handled by NSGA-II.

In Fig. 6 each candidate (red points) represents a channel combination that was used for obtaining the sub-dataset and use it as input to EEGNet. The *best points* that appear in the Pareto-front (green points) represent the maximum accuracies that can be reached with that number of channels. For example, using EEG data from *low vs high arousal* of subject 1 and four channels, the maximum accuracy that can be reached by EEGNet is 0.800.

Tables 3 and 4 present the accuracies of the channel combinations in the Pareto-front handled by the NSGA-II algorithm and classified by EEGNet for all the subjects, for *low vs high arousal/valence classification*, respectively. Since our objective is the optimal reduction of the number of channels used in the classification process, and because the maximum accuracies are reached using fewer than 15 channels in most of the cases, the tables only present the accuracies using the set of 1–15 channels in the Pareto-front.

**Table 3:** Low vs high arousal classification accuracies obtained with EEGNet.

S	Channels														
	1	2	3	4	5	6	7	8	9	10	11	12	13	14	15
1		0.751		0.800	0.867				0.926	0.940				0.954	0.979
2	0.729	0.831	0.893	0.911	0.929	0.942		0.969							
3	0.726	0.830		0.867		0.911	0.93		0.933		0.948	0.952		0.959	
4	0.733	0.796	0.867		0.896		0.921	0.925		0.929					
5	0.733	0.738	0.767	0.838			0.871	0.875	0.896				0.900	0.921	
6	0.727	0.767	0.800	0.893		**0.967**	**0.987**	**1.000**							
7	0.717	0.739	0.817	0.867	0.883		0.917	0.944	0.972			0.989			
8	0.504	0.693	0.785	0.819	0.878	0.889		0.900		0.922	0.941		0.956		
9	0.716	0.786	0.821	0.825	0.888		0.909	0.961			0.968		0.986		
10	0.803	0.843	0.890	0.907	0.913	0.927		0.93	0.95	0.953	0.973				
11	0.679	0.788	0.838		0.883		0.946				0.958	0.962		0.975	0.992
12		0.653	0.789	0.853	0.87	0.912	0.93	0.954		0.968				0.972	
13	0.752	0.796	0.822	0.874	0.893	0.919	0.93	0.948							
14	0.70	0.797	0.813	0.873		0.893	0.91	0.917	0.93		0.96	0.967			
15	0.683	0.82	0.850			0.86	0.88	0.883	0.910						
16	0.711	0.742	0.778	0.818	0.827		0.853	0.889			0.933		0.969		
17	0.644	0.719	0.774		0.833		0.881	0.915			0.978		0.989		
18	0.614	0.876	0.890	0.914		0.924	0.933	0.952	0.962		0.976				
19	0.722		0.769	0.796	0.859	0.894				0.925	0.933			0.937	
20	0.722	**0.886**		**0.933**	0.945	0.961	0.98				0.984				
21		0.681	0.786	0.814	0.818	0.867	0.891		0.909			0.947		0.954	0.968
22	0.667	0.823				0.830							0.837		
23	0.744	0.851	0.887	0.908		0.913		0.949	0.985					0.99	1.000
24	0.533	0.709	0.807	0.842	0.881		0.895		0.898	0.902	0.905		0.909		
25		0.723	0.814	0.825	0.856		0.884	0.902			0.909		0.926		0.933
26	0.700	0.771	0.838	0.857	0.886	0.914	0.919	0.952							
27	**0.867**		**0.933**		**0.947**	0.967	0.987	0.993			1.000				
28	0.747	0.760	0.844	0.907	0.942			0.951	0.964				0.978		0.982
29	0.667		0.733	0.804		0.855	0.882		0.886	0.91				0.925	
30	0.718	0.790	0.805	0.851	0.903	0.928	0.985		0.99	0.995					
31	0.752	0.786	0.824	0.886	0.914		0.933		0.948	0.957	0.967				
32	0.600	0.750	0.797	0.89						0.917				0.923	0.967

Accuracies obtained in the Pareto-front for the first 1–15 channels selected by NSGA-II.

The subjects with the highest accuracies per channel set are indicated in bold.

**Table 4:** Low vs high valence classification accuracies obtained with EEGNet.

S	Channels														
	1	2	3	4	5	6	7	8	9	10	11	12	13	14	15
1	0.812	0.929	0.933	0.938	0.946	0.975		0.992		1.000					
2	0.592	0.721	0.792	0.833	0.871	0.875	0.879	0.908	0.917						
3	0.733	0.833	0.967	0.975	**1.000**										
4		0.792	0.825	0.838	0.875	0.9	0.908		0.921			0.938		0.942	
5	0.558	0.649	0.814	0.86	0.874	0.881	0.909	0.94							
6	0.698		0.827	0.867	0.882	0.933		0.941			0.976		0.980		0.984
7	0.564	0.76	0.796	0.911			0.964					0.978	0.991		1.000
8	0.650	0.675	0.717	0.783	0.838		0.862	0.912	0.929	0.938					
9	0.693	0.778	0.818	0.84		0.849	0.862		0.876	0.884	0.920				
10	0.589	0.674	0.785	0.800	0.852			0.878		0.915		0.933		0.944	
11	0.644	0.693	0.804	0.844	0.849	0.871		0.911							
12	0.905	0.962	**0.981**	**1.000**											
13	**0.922**	**1.000**													
14	0.615	0.738	0.774		0.826	0.831		0.856	0.872	0.908		0.928			
15	0.775	0.867	0.898		0.916		0.94	0.979							
16	0.800	0.890	0.900	0.910	0.930		0.943		0.96	0.977		0.983	0.997		
17	0.716		0.738	0.751	0.756		0.778			0.782	0.800				
18		0.791	0.844	0.849	0.88		0.893		0.907			0.924			0.929
19	0.554	0.744	0.867		0.877	0.913	0.938	0.944	0.954						
20	0.822	**0.904**	0.941	0.956	0.978	1.000									
21	0.633	0.825	0.933	0.958	0.975		0.983		1.000						
22	0.684	0.809	0.831	0.858	0.871	0.876	0.889	0.907	0.916						
23	0.641	0.754	0.81	0.846	0.892			0.923			0.944	0.969		0.974	0.979
24	0.648	0.876	0.914	0.971		0.981	1.000								
25	0.747	0.793	0.847	0.940	0.993	1.000									
26	0.569	0.725	0.839	0.863	0.914	0.929	0.957	0.984	0.996						
27	0.692	0.856	0.903		0.918	0.949	0.985	0.990		0.995					
28	0.660	0.737	0.787			0.803	0.807	0.823	0.903	0.907					0.930
29	0.621	0.713	0.774	0.790	0.841		0.877	0.985					0.995		
30	0.627	0.740		0.777	0.84		0.860	0.883	0.940						
31	0.713	0.760	0.843		0.867	0.877	0.917	0.947		0.960			0.977		
32	0.772	0.856	0.928	0.967	0.994	1.000									

Accuracies obtained in the Pareto-front for the first 1-15 channels selected by NSGA-II.

The subjects with the highest accuracies per channel set are indicated in bold.

Looking at the average accuracies obtained using all channels and 2-second segments (see left part of Fig. 3), the accuracy was around 0.930, and using fewer channels selected by NSGA-II for some subjects there are channel combinations which can obtain accuracies up to 1.000 with 8 channels.

**Figure 8:**
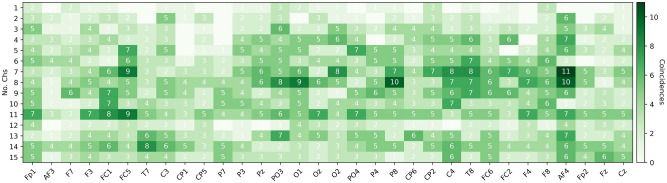
EEG channels selected for 2-s segments classification of low and high arousal using EEGNet and NSGA-II.

**Figure 9:**
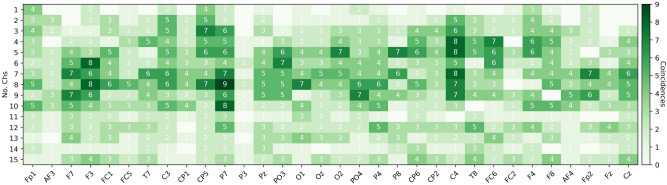
EEG channels selected for 2-s segments classification of low and high valence using EEGNet and NSGA-II.

To explore if there exist a common set of selected channels or a channel distribution pattern across subjects, Figs. 8 and 9 present the subsets with 1–15 channels used to obtain the highest accuracies (the results in the Pareto-front) for *low vs high arousal/valence* classification, respectively, using EEGNet.

The results indicate the coincidences of a given channel selected across subjects, for each of the first 15 sets in the Pareto-fronts. For example, Fig. 8 shows that when the set of selected channels in the Pareto-front was 1, the channel *Fp1* was used by 2 of the 32 subjects, and *PO3* by three subjects. In this regard, Fig. 8 shows that the channels with more coincidences among subjects occur when the set of channels in the Pareto-front contain 7–11 channels.

**Figure 10:**
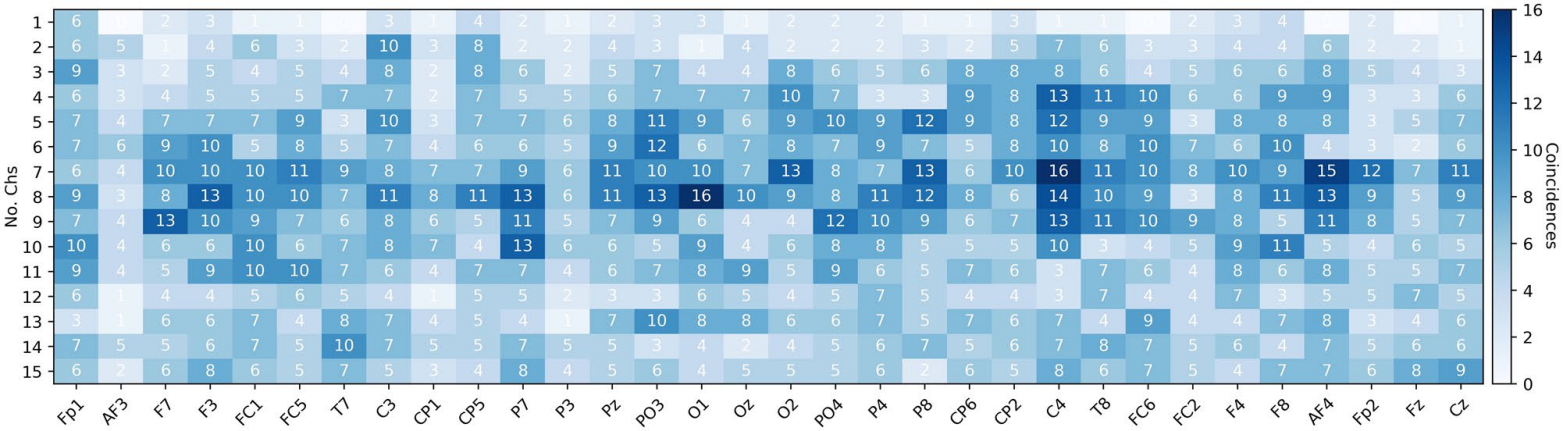
EEG channels selected for classification of both low and high arousal and valence using EEGNet and NSGA-II.

Examining these figures one can argue that there are some important channels, since they were used in the selected sets for about 35% of the subjects. For example channel *AF4* is one of the most used channels for *low vs high arousal* classification according to Fig. 8, but for *low vs high valence* it is not, instead *C4* is one of the most used, as it is shown in Fig. 9.

The set of experiments exposed have been carried out using one dimension at the time, Arousal or Valence. However if we are interested in finding a unique set of channels for both dimensions, we can use the chromosomes generated by NSGA in each iteration for the classification of both dimensions in parallel or simultaneously. This may reduce the accuracies, since the algorithm will be forced to select the same channels for both tasks.

To provide a first overview of the most relevant channels for both tasks, Fig 10 presents the coincidences among subjects and dimensions for the 1–15 set of selected channels by selected by NSGA-II. This can give us an impression of the most used channels, but also of the less used channels. Some of the clearly most used channels are *O1, C4, AF4*, and the less used are *AF3, P3, Fz*. When we used only 1 channel for classification of *low vs high arousal/valence* the highest coincidence is 6, which correspond to two subjects for arousal and four subjects for valence.

The less used channels are consistent across all the sets (1–15), which means that they were used for only a few subjects or the channels were not part of the Pareto-fronts of the subjects. Another interesting point is that the highest coincidences occurred when using 4–10 channels in the sets, which is when the highest accuracies were reached (see Table 3 and 4).

## Discussion

We presented a set of experiments where we first tested our proposed methods for feature extraction based on DWT and classification using SVM, NB, and kNN^57,59,63^. The results using all the channels and creating one model per subject suggest that this approach is not suitable for the task, since the accuracy in the best case was 0.687 and 0.561 in the worst.

From this insight, we have implemented a CNN-based method for EEG-based Low vs High Arousal/Valence classification using EEGNet. We performed experiments using all the available channels with the CNN, as well as using one channel at a time, this with the aim of comparing the results with state-of-the-art proposals. After this, we performed the experiments using the CNN combined with NSGA-II for channel selection for both, Arousal and Valence dimensions. Experimental results show that we can differentiate between Low and High Arousal/Valence with higher accuracy, while at the same time reducing the number of required EEG channels from 32 to a subset lower than 10 and obtain similar or higher classification accuracies.

**Figure 11:**
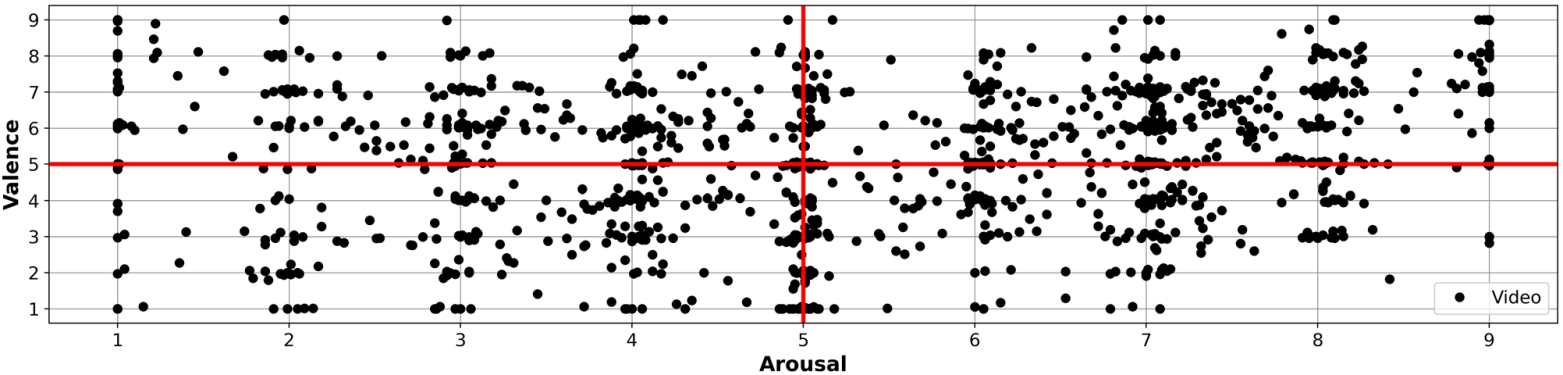
Distribution of arousal and valence rating values for the videos presented to all the subjects in the DEAP dataset.

The results obtained are encouraging and indicate that it is possible to identify with high accuracy when a subject reported Low or High Arousal/Valence using a few electrodes, even using very close rating values from low and high (see Fig. 11).

In the first experiment using CNN and all the available channels, the highest accuracies were obtained using 2-s segments. Because of this, and because the computational cost of the CNN, we performed the experiments with only 2-s segments. However, further exploration about common channels among the use of different signal segments should be performed, to ensure the relevance of certain channels for both, *low vs high arousal* and *low vs high valence*.

As it has been shown in the experiments using different EEG signal segments size we can increase the number of instances easily. The experiments were performed using 2-s segments, however future experiments will consider the creating of overlapping window instances and analyze if the performance improves, taking care of over-fitting and bias-error that this may produce. Following the overlapping window approach we will also test if increasing the number of instances with 5- and 10-s segments helps to increase the performance.

As it was presented in Figs. 8, 9, and 10, there are some channels that were selected by NSGA-II in different Pareto-fronts, which indicated that the channels are relevant for both task. The design of a personalized low-density EEG headset can be the best adoption approach, but this results also indicates that we can find some relevant common channels and create (or calibrate) a single EEG headset when required. One way to do this is by forcing the NSGA-II algorithm to select the best channels to classify *low vs high arousal* and *low vs high valence* at the same time. In this case, the generated chromosomes in each population must be tested across all subjects. This means that we have to optimize 67 objectives; increase the classification accuracy of each of the 32 subjects for Arousal and Valence (66 objectives), and decrease the number of channels (1 objective). Alternatively, we can also simply optimize three objectives; increase the mean classification accuracy of all the subjects for Arousal and Valence (2 objectives), and decrease the number of channels (1 objective).

Other approaches have considered Low Arousal/Valence from 1 to 4.8 rating values, and High Arousal/Valence as 5.2–9 rating values, which may help to increase the performance, removing the EEG instances corresponding to the rating values from 4.8 to 5.2^39^. This will be considered for future work, specially for cross-subject models, since the SAM feedback may vary between subjects and this may help to unify it.

Similar studies have presented NN architectures for extracting the most relevant features and classification of emotions, validated in various private and public datasets^64–69^, based on those proposals, our future work will consider to combine some parts for pre-processing, feature extraction and classification of emotions. For instance, we could compare if decomposing the data into sub-bands using a different approach than DWT or the Empirical Mode Decomposition (EMD)^57,70^, or using methods such as common spatial pattern (CSP) yields more useful information^71^.

In general, we will continue testing NN-based methods for handling the whole process, as well as already described emotion-related features for improving the classification performance, and thus help the proposed NSGA-based algorithm to select the most relevant channels^57,64–71^. Future work will also be pointed to finding the best way and test the effectiveness for cross-subject models using CNN as well as testing our previous proposals using DWT or EMD for feature extraction^53,57,59,63^.

The proposed method was applied to the DEAP dataset, which is one of the most used for two-dimensional emotion classification. After analyzing the protocol for emotion elicitation and feedback collection, our future work will be focused on proposing a new protocol using the well-accepted International Affective Picture System (IAPS) and collecting the feedback using the SAM approach.

The results obtained using CNN instead of DWT-based features and classical machine learning appears to be more promising. The problem is that CNNs are computationally expensive. For this specific application the models can be trained using data collected beforehand, and used the created models later, once the model is trained the required time to classify a new instance is the same or similar than a traditional machine learning algorithm, so it does not affect an application for real-time detection of emotions. Future steps will focus on finding a way to reduce the required layers of the CNN-architecture, and improved it to extract more features in frequency and amplitude domain, as well as for selecting the most relevant sub-bands associated with the elicited emotions.

## Methods

### DEAP dataset, pre-processing, feature extraction and classification using EEGNet

The DEAP dataset was collected from 32 subjects (16 males, 16 females) with mean age 26.9 using 32 active AgCl electrodes located according to the 10–20 international system, and a sample rate of 512 Hz.

According to the authors of the DEAP dataset, each participant signed a consent form and filled out a questionnaire prior to the experiment^2^. All the procedures were performed in accordance with the relevant guidelines and regulations or in accordance with the Declaration of Helsinki.

The protocol followed for stimulating and collecting the EEG signals consisted on presenting 40–60-s music videos. The experiment session started with a two-minute baseline recording and the subjects were asked to relax. Then, the process for displaying each of the 40 music video consisted of four steps: (1) a 2-s screen displayed the current trial number, (2) a 5-s baseline recording, (3) the 60-s music video is presented, and (4) the subject rated the music video terms of *valence, arousal, like/dislike,* and *dominance*^2^.

Figure 11 presents the distribution of the Arousal and Valence rating values of all the videos presented to the subjects in the DEAP dataset. The red lines indicate the low and high values separation, for instance, if the Arousal value is $<5$ is it assigned as *LowArousal*, otherwise *HighArousal*, and the same for Valence.

As it is shown in Fig. 11, the red lines indicating the separation of classes contain rating values of several videos, specially to separate *LowArousal* and *HighArousal*. For the experiments exposed here we did not remove any of the instances, since in this way it can be compared with other approaches and future improvements of this approach. However, if we remove the closest values to the red line, the classification accuracies will possibly increase.

The EEG signals from the DEAP dataset were down-sampled to 128 Hz, EOG artifacts were removed, then a band-pass frequency filter from 4-45 Hz was applied. Finally, the CAR method was also applied^2^. We separated the 60-s segments corresponded to the exposure of the music videos ($128*60 = 7680$ data points), and depending on the experiment, as presented in Table 1, the EEG signal segments were separated into segments of 2, 5, and 10 s. Depending on the experiment, the EEG signal segments were used as input to the EEGNet.

EEGNet is a compact CNN architecture for EEG signal processing and classification implemented on *python Keras* by the Army Research Laboratory (ARL)^62^. It has been tested for different EEG-based task classification and trained with limited data, and it has shown higher accuracies than some ML-based classifiers^43,45,62,72^.

**Figure 12:**
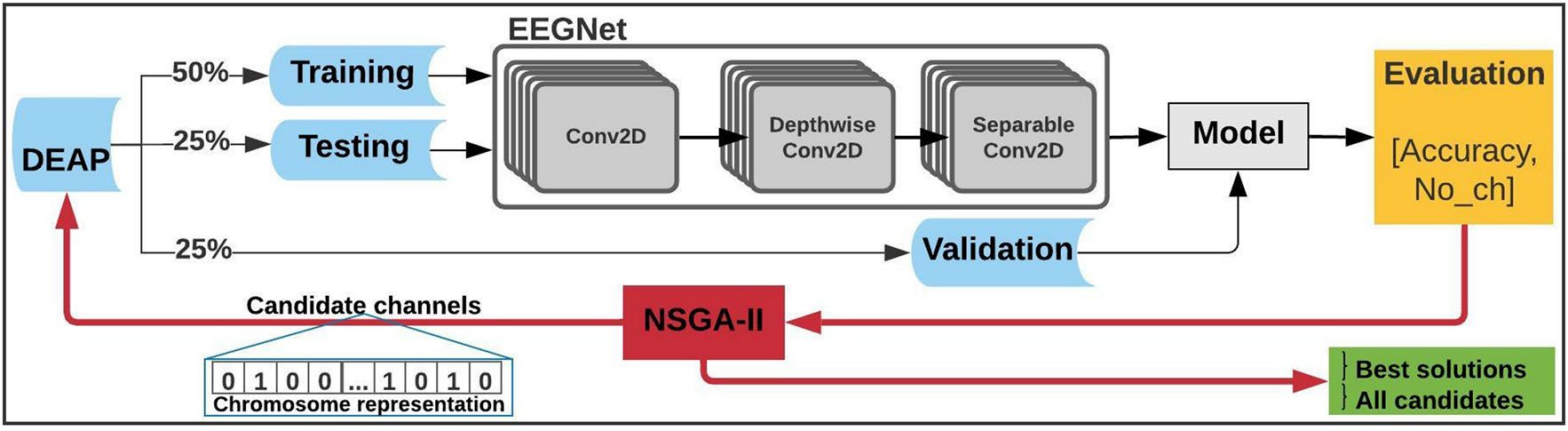
Flowchart of the optimization process for EEG channel selection using a chromosome representation for NSGA-II.

As it is illustrated in Fig. 12, the CNN architecture consist of a *2D convolutional filter*, a *Depthwise convolution*, and a *Separable Convolution*, which can be summarized as follows: *Block 1* perform two convolutional steps in sequence. First, it fit’s a *2D convolutional filter*, with the filter length chosen to be half the sampling rate, resulting in feature maps that contain different band-pass frequencies of the EEG signal. Then, a *Depthwise convolution* that learns a spatial filter, is applied. It applies a *Batch Normalization* along the feature map dimension before applying the *exponential linear unit (ELU)* nonlinearity, and to help regularize it uses the *Dropout* technique. After that, it applies an *average pooling* layer to reduce the sampling rate, and regularize each spatial filter by using a maximum norm constraint of 1. *Block 2* uses a *Separable Convolution*, which is a *Depthwise Convolution* followed by *Pointwise Convolutions*. Then an *Average Pooling* layer for dimension reduction is used. *Last block*, the features are managed by a *softmax* classification with N units, where N is the number of classes.

### Optimized EEG channel selection process

Channel selection process is an important step for decreasing the computational cost of any DL/ML algorithm, and with this reach cheaper low-density EEG headset. More importantly, selecting a set of channels will allow to focus on the most relevant information or brain area, this will contribute to increase or maintain the classification accuracy using DL/ML. For this, we have continued our research using genetic algorithms (GAs) and multi-objective optimization (MOO) algorithms.

For channel selection, we have applied an NSGA-based process, which uses a non-dominated sorting ranking selection method to emphasize good candidates and a niche method is used to maintain stable sub-populations of good points^58^. Specifically, we have used NSGA-II since it has proven to find the most relevant channels for different EEG-based applications with 2–3 objectives^52,53,59^. NSGA-II solved certain problems related to the computational complexity, non-elitist approach, and the need to specify a sharing parameter to ensure diversity in a population presented in the first version. It reduced the computational cost from $O(MN^{3})$ to $O(MN^{2})$, where *M* is the number of objectives and *N* the population size. It also introduced an elitist approach by comparing the current population with the previously found best non-dominated solutions^73^.

The problem to be optimized, which is illustrated in the flowchart of Fig. 12, is defined by two unconstrained objectives based on NSGA-II structure; (1) decrease/select the number of required and most relevant EEG channels for classifying *low vs high arousal/valence*, while (2) increasing or at least maintaining the EEGNet-based classification accuracy. For this, we organized the DEAP dataset, each segment-size case separately, and used a chromosome to represented the 32 EEG channels of the solution domain using binary values, where each gene in the chromosome represents an EEG channel; 1 if the EEG channel is used in the classification process and 0 if not (see chromosome representation or candidate channels in Fig. 12).

NSGA-II uses a fitness function to evaluate the solutions domain of the two-objective optimization problem, which in this case is defined as [*Acc*, *No*], where *Acc* is the EEGNet-based classification accuracy obtained with each chromosome in each population and *No* the number of EEG channels used, which are the ones indicated with 1 in the chromosome.

The optimization process handled by the NSGA-II algorithm starts by creating the possible candidates or chromosomes in the population, which represent an iteration of NSGA-II. It obtains the corresponding raw EEG data for the channels represented as 1 in each chromosome, and then we create an EEGNet Model using $50\%$ of the data, $25\%$ for testing, and $25\%$ for validating the created model. The obtained accuracy and the number of EEG channels used ([*Acc*, *No*]) is returned to the NSGA-II to evaluate each chromosome in the current population. The process is repeated creating populations of 10 chromosomes, which was determined experimentally. The termination criterion for the optimization process is defined by the objective space tolerance, which is defined as 0.001, this criterion is calculated every 10*th* generation. If optimization is not achieved, the process stops after a maximum of 100 generations, which is also determined experimentally.

## Data Availability

The DEAP dataset used for this study is publicly available and it can be found at eecs.qmul.ac.uk/mmv/datasets/deap The method for channel selection using NSGA is publicly available at github.com/wavesresearch/MOO_ch_selection_DEAP.
